# Inhibition of *Candida albicans* Biofilm Formation and Attenuation of Its Virulence by *Liriope muscari*

**DOI:** 10.3390/antibiotics13050434

**Published:** 2024-05-12

**Authors:** Jeonghoon Lee, Hyunchan Song, Kiyoung Kim

**Affiliations:** 1 Department of Medical Science of Meridian, College of Korean Medicine, Graduate School, Kyung Hee University, Kyungheedae-ro 6-gil, Dongdaemun-gu, Seoul 02447, Republic of Korea; ljh@boguclinic.com; 2 Graduate School of Biotechnology, Kyung Hee University, 1732, Deogyeong-daero, Giheung-gu, Yongin-si 17104, Republic of Korea; songhchan@naver.com

**Keywords:** *Candida albicans*, *Liriope muscari*, biofilm formation, antifungal agent, dimorphic transition, adherence assay

## Abstract

(1) Background: Although *Candida albicans* accounts for the majority of fungal infections, therapeutic options are limited and require alternative antifungal agents with new targets; (2) Methods: A biofilm formation assay with RPMI1640 medium was performed with *Liriope muscari* extract. A combination antifungal assay, dimorphic transition assay, and adhesion assay were performed under the biofilm formation condition to determine the anti-biofilm formation effect. qRT-PCR analysis was accomplished to confirm changes in gene expression; (3) Results: *L. muscari* extract significantly reduces biofilm formation by 51.65% at 1.56 μg/mL use and therefore increases susceptibility to miconazole. *L. muscari* extract also inhibited the dimorphic transition of Candida; nearly 50% of the transition was inhibited when 1.56 μg/mL of the extract was treated. The extract of *L. muscari* inhibited the expression of genes related to hyphal development and extracellular matrix of 34.4% and 36.0%, respectively, as well as genes within the Ras1-cAMP-PKA, Cph2-Tec1, and MAP kinase signaling pathways of 25.58%, 7.1% and 15.8%, respectively, at 1.56 μg/mL of *L. muscari* extract treatment; (4) Conclusions: *L. muscari* extract significantly reduced Candida biofilm formation, which lead to induced antifungal susceptibility to miconazole. It suggests that *L. muscari* extract is a promising anti-biofilm candidate of *Candida albicans* since the biofilm formation of *Candida albicans* is an excellent target for candidiasis regulation.

## 1. Introduction

*Candida albicans* accounts for the majority of fungal pathogens found and has a mortality rate of 40–60% [1,2,3]. It is typically found in the mouth, skin, and intestines. Sometimes, it can invade the human body, causing infection in the blood, bones, brain, and heart [2]. The annual expenditure for the treatment of fungal diseases was $4.5 billion in the United States alone, that for Candida infections cost the most with $1.4 billion, while that for aspergillus infections cost $1.2 billion [2,3,4]. Additionally, there were 3,639,037 candidiasis outpatient visits, costing $1.6 billion out of the total outpatient costs of $2.6 billion. The second-highest outpatient cost was for patients with dermatophyte infections, amounting to $802 million [4]. 

Recently, efforts to discover alternative methods for eradicating biofilm-related infections have increased [5]. Early antibiotics caused an increased resistance of fungi [6]. One of the new therapeutic approaches to medication for fungal infections is combinational therapies [5]. It targets several aspects of micro-organisms to control infections. Biofilm formation of pathogenic fungi is now at the center of attention due to their increasing resistance to commercially available antifungal agents and environmental conditions [7,8,9].

*C. albicans* biofilm formation involves a complex developmental process. It begins with the attachment of planktonic cells to a substrate, followed by cell proliferation, hyphal growth, and extracellular matrix production [10,11,12]. Eventually, mature cells disperse from the biofilm into the surrounding environment [10]. Planktonic cells are mostly vulnerable to several agents since there are no defensive barriers between the cells and the environment [13]. The biofilm formation is a critical step in pathogenesis because *C. albicans* tends to exhibit reduced sensitivity or insensitivity to antifungal agents [14,15].

Natural substances significantly contribute to the discovery and development of drugs [16,17]. More than 75% of the treatment drugs used against infectious diseases are derived from nature [18]. It has advantages over synthetic medications in cost and the number of potential candidates [19]. Also, it may lead to shortcuts to study combinational therapies [5].

The genus *Liriope* has been pharmacologically used in several Asian countries. It was historically proven to treat various diseases [20]. With the advancement of technologies, recent studies analyzed the chemicals of genus *Liriope* involved in traditional use [20,21]. *Liriope muscari*, one of the genus *Liriope* plants, has been utilized in traditional Chinese medicine for the treatment of various ailments including pharyngitis, bronchitis, pneumonia, cough, and cardiovascular disease [22]. Previous studies found that they have novel bioactive compounds including steroidal saponins, flavonoids, and sesquiterpene glucoside [20]. For example, a novel steroidal saponin DT-13 isolated from *L. muscari* reduced the adhesion of human breast cancer cells and their migration under hypoxia [23,24]. However, neither the activities of the whole extract of *L. muscari* nor the compounds in them against *C. albicans* have been studied. It means that the inhibition of attachment to the animal cells or the surrounding area, transitions from yeast to hyphae, and biofilm formation of *C. albicans* using *L. muscari* remain largely unclear. The objectives of this study were to (i) evaluate the potential inhibition by *L. muscari* on biofilm formation of *C. albicans* and (ii) improve the understanding of *L. muscari* triggering anti-biofilm mechanisms. 

## 2. Results

### 2.1. Anti-biofilm effect of L. muscari

**Figure 1:**
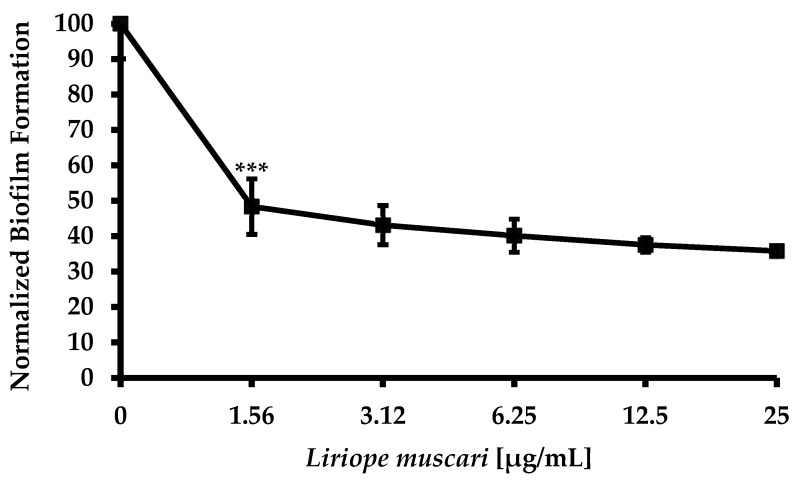
*L. muscari* extract inhibited biofilm formation. (***: *p*-value < 0.001).

*L. muscari* was treated for the purpose of determining its anti-biofilm activity. *L. muscari* significantly reduced biofilm density in the strains tested (Figure 1). The IC_50_ value of *L. muscari* for inhibition of biofilm formation was nearly 1.56 μg/mL against *C. albicans* (Figure 1). With only the use of 1.56 μg/mL, 51.65 ± 7.83% of biofilm formation was inhibited.

### 2.2. L. muscari Extract Increased the Susceptibility of Commercial Antibiotics to C. albicans

**Figure 2:**
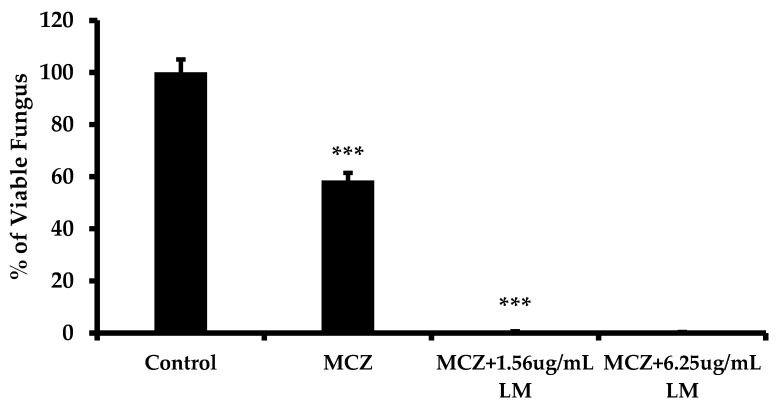
*L. muscari* increased the susceptibility of MCZ to *C. albicans*. Biofilms of *C. albicans* were formed after 24 h of incubation in YPD followed by 24 h of treatment with MCZ (3.125 μg/mL) alone or in combination with *L. muscari* (1.56 and 6.25 μg/mL). MCZ: Miconazole, LM: *L. muscari* extract. (***: *p*-value < 0.001).

*L. muscari* extract was combined with antibiotics to investigate if inhibiting biofilm formation would influence antibiotic susceptibility. After 24 h of growth, Candida cultures were treated with the indicated concentrations of miconazole (MCZ) with or without *L. muscari* (LM) extract for an additional 24 h. *L. muscari* extract increased susceptibility to antifungal agents (Figure 2). When MCZ was treated alone, 58.53 ± 2.92% of viable fungus remained. However, when *L. muscari* was treated with MCZ, a reduced amount of MCZ use for *C. albicans* control was observed, which means L. muscari-induced susceptibility of *C. albicans* on MCZ had increased.

### 2.3. Inhibitory Activity of L. muscari on C. albicans Dimorphic Transition from Yeast to Hyphae

**Figure 3:**
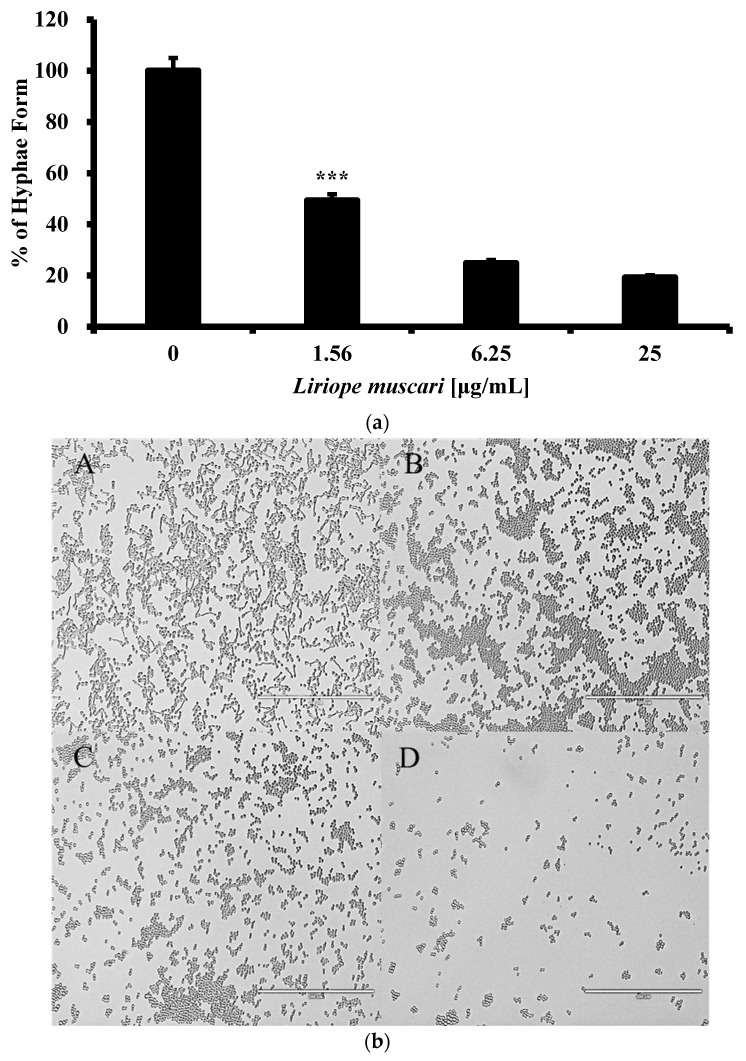

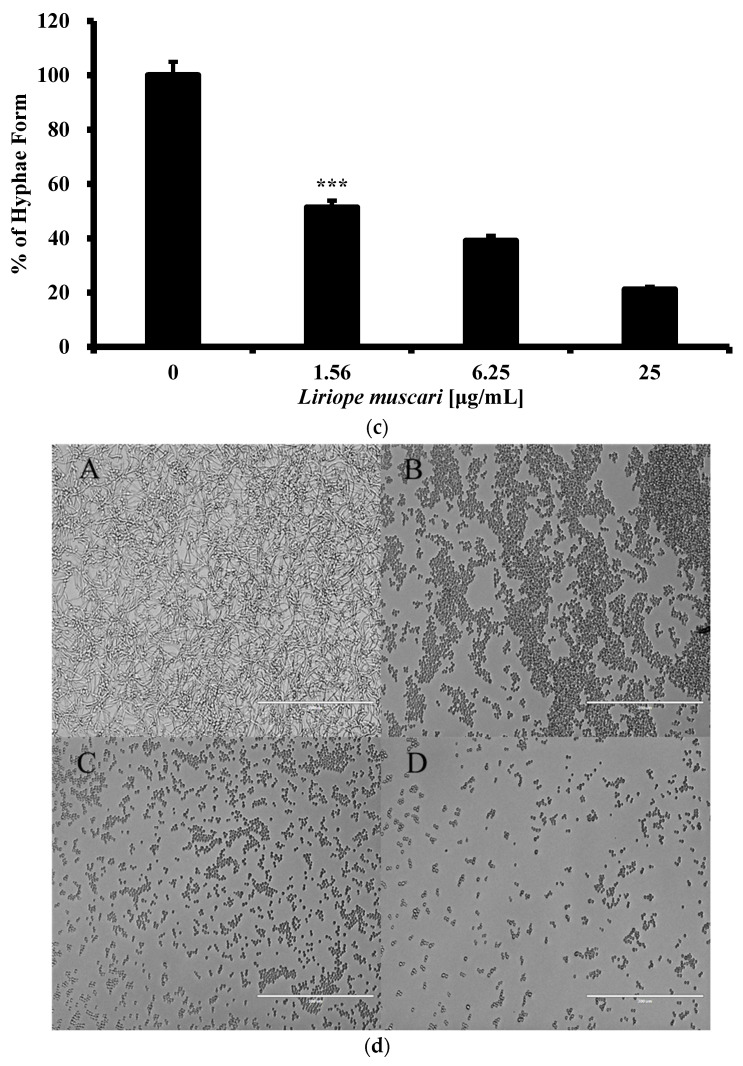
*L. muscari* inhibited the transition of *C. albicans* from yeast to hyphae. (**a**) *L. muscari* inhibited *C. albicans* filamentation induced in RPMI 1640. *C. albicans* (1 × 10^6^ cells/mL) and *L. muscari* (1.56, 6.25, and 25 μg/mL) were incubated at 37 °C for 4 h. (**b**) Images of *C. albicans* cells grown in RPMI 1640. *L. muscari* was added at concentrations of 1.56, 6.25, and 25 μg/mL. A: RPMI 1640 control, B: *L. muscari* 1.56 μg/mL, C: *L. muscari* 6.25 μg/mL, D: *L. muscari* 25 μg/mL. (**c**) *L. muscari* inhibited *C. albicans* filamentation induced in 10% FBS YPD medium. *C. albicans* (1 × 10^6^ cells/mL) and *L. muscari* (1.56, 6.25, and 25 μg/mL) were incubated at 37 °C for 4 h. (**d**) Images of *C. albicans* cells incubated in 10% FBS YPD medium. *L. muscari* was added at concentrations of 1.56, 6.25, and 25 μg/mL. A: 10% FBS YPD control, B: *L. muscari* 1.56 μg/mL, C: *L. muscari* 6.25 μg/mL, D: *L. muscari* 25 μg/mL. Scale bar: 200 μm. (***: *p*-value < 0.001).

Inhibition of *C. albicans* dimorphic transition by *L. muscari* was determined. Since not only can nutrient-deficient spider medium but also liquid RPMI 1640 and mammalian serum trigger the formation of hyphae, we conducted the examination using both conditions [25]. At a low concentration of 1.56 μg/mL, *L. muscari* effectively interrupted hyphal formation in RPMI 1640 or 10% FBS YPD medium to 49.3 ± 2.4% and 51.3 ± 2.5%, respectively (Figure 3a–d). Therefore, these results suggest that *L. muscari* might have the ability to impede the transition from yeast to the hyphal form of *C. albicans*.

### 2.4. Suppression of the Gene Expression Related to Biofilm Formation Regulatory Proteins after L. muscari Treatment

To analyze the molecular background of *L. muscari*’s suppression of hyphal growth and cell attachment, qRT-PCR was conducted to determine the expression of genes involved in hyphal growth and cell attachment in *C. albicans*. The planktonic cells were included in the process to determine whether those changes were caused within the hyphal growth.

**Figure 4:**
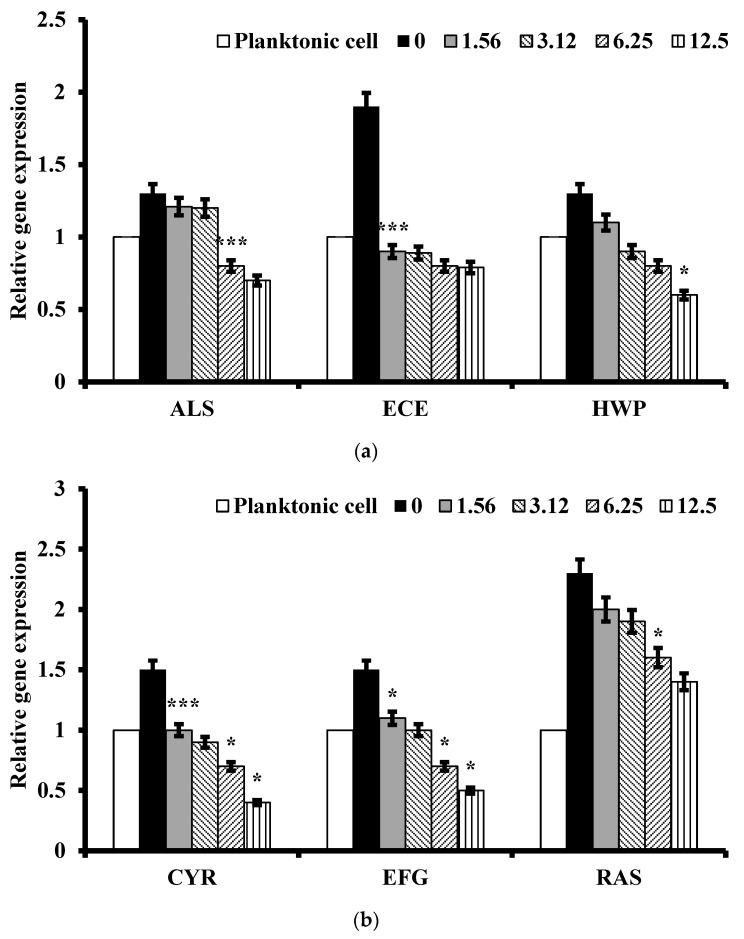

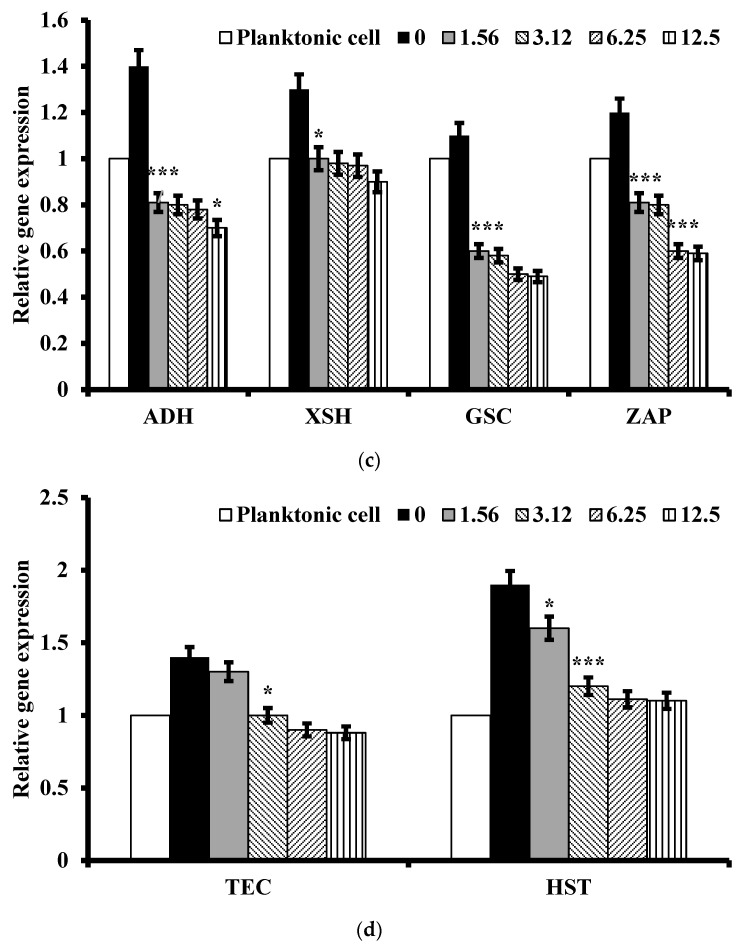
*L. muscari* decreased the expression of genes for hyphae-specific regulation, extracellular matrix, Ras1-cAMP-Efg1, MAP kinase, and Cph2-Tec1 in *C. albicans*. (**a**) Relative expression of genes regulating hyphae-specific genes. (**b**) Relative expression of genes regulated by the Ras1-cAMP-Efg1 pathway. (**c**) Relative expression of genes regulating extracellular matrix genes. (**d**) Relative expression of genes related to the Cph2-Tec1 pathway and the MAP kinase pathway. Total RNA was extracted from *C. albicans*, including control and biofilms treated with the indicated concentrations of *L. muscari*, synthesized cDNA, and examined by qPCR using the corresponding primers. (*: *p*-value <0.05, ***: *p*-value < 0.001).

After treatment with 1.56–25 μg/mL of *L. muscari*, the expression levels of four genes (RAS1, EFG1, TEC1, and CDC35) associated with the Ras1-cAMP-Efg1 pathway [26] were significantly reduced (Figure 4a–d). Ras1p is a GTPase that acts to induce the formation of hyphae by activating both the MAPK cascade pathway and the Ras1-cAMP-Efg1 pathway [27]. Efg1p is a transcription factor of the Ras1-cAMP-Efg1 pathway, which is important in regulating the expression of certain hyphae-specific genes, including ECE1, HWP1, and ALS3 [28].

**Figure 5:**
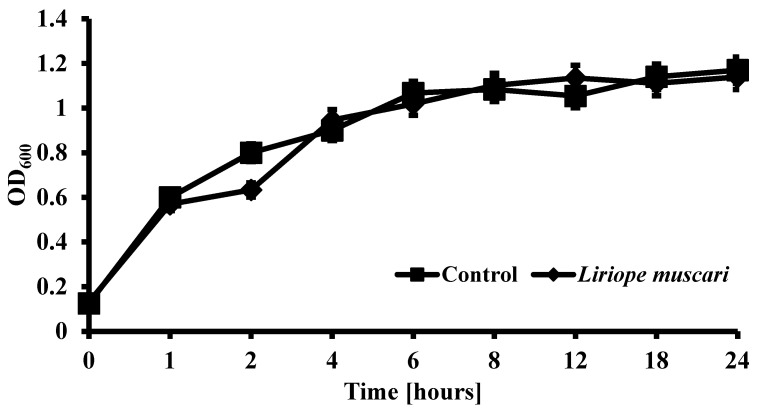
*L. muscari* lacks the ability to inhibit the growth of *C. albicans*.

*L. muscari* caused alterations in the transcription level of 12 genes involved in biofilm formation which were evaluated by qRT-PCR. The degree of gene expression for each gene was analyzed in cells treated with *L. muscari* (1.56–12.5 μg/mL) for 24 h and compared to untreated cells and planktonic cells (Figure 5). Results showed that *L. muscari* significantly repressed the expression levels of the following genes: hypha essential genes (ALS3, ECE1, and HWP1), Ras1-cAMP-Efg1 pathway (CYR1, EFG1, and RAS1), extracellular matrix (ADH5, CSH1, GSC1, and ZAP1), Cph2-Tec1 pathway (TEC1), and MAP kinase pathway (HST7). The expression of the genes related to hyphal development were decreased by 34.4%, and those of the extracellular matrix were decreased by 36.0% at 1.56 μg/mL. Also, the genes within the Ras1-cAMP-PKA, Cph2-Tec1, and MAP kinase signaling pathways were downregulated by 25.58%, 7.1%, and 15.8%, respectively, in the same concentration.

### 2.5. L. muscari Had No Effect on Sum of Fungal Growth

To determine whether the inhibition of biofilm formation by *L. muscari* resulted from its antifungal activity, *C. albicans* was exposed to specified concentrations of *L. muscari* for 24 h. Surprisingly, there were no clear differences between the treatment and the control. Both groups exhibited the growth of *C. albicans* in a time-dependent manner, which means *L. muscari* only affects the biofilm formation of *C. albicans* while not affecting fungal growth (Figure 5).

## 3. Discussion

*C. albicans* is a yeast that opportunistically causes systemic infections in individuals with compromised immune systems [2]. Nosocomial *C. albicans* infections are frequently associated with their capacity to produce biofilms on mucosal surfaces and medical devices [3,29,30]. The formation of biofilms is a finely regulated process, governed by multiple interconnected signaling pathways [25], resulting in structured microbial communities attached to surfaces and enclosed in an exopolymeric extracellular matrix [3,29,31].

The formation of biofilms is a critical stage in pathogenesis, as *C. albicans* within biofilm are generally less susceptible or resistant to antifungal agents [32]. Consequently, the development of new antifungal agents with inhibitory activity on the attachment of *C. albicans* attachment, the transition of yeast-to-hyphae, and biofilm formation should be an important strategy.

In this study, *L. muscari* was examined for an inhibitor of biofilm formation by *C. albicans*. *L. muscari* also increased antifungal activity by inhibiting biofilm formation (Figure 2). Since infections caused by micro-organisms are hard to cure due to their adaptations to antibiotics and their rapid mutations [33], *L. muscari* can be useful as a great adjuvant when used with the usual antibiotics as we found in this study. Additionally, we discovered that *L. muscari* could potentially make *C. albicans* more susceptible to other chemicals by inhibiting biofilm formation. This is expected to enhance the cost-effectiveness of the currently used treatment.

*L. muscari* at 6.25 μg/mL inhibited the transformation of hyphal morphology in liquid RPMI 1640 and 10% FBS YPD medium (Figure 3). This indicates the potential application of *L. muscari* as an agent to counteract the virulence of *C. albicans*.

In an effort to understand the mechanisms responsible for the anti-biofilm effects of *L. muscari* on *C. albicans*, we analyzed the gene expression profiles related to cell adhesion, hyphal development, and extracellular matrix synthesis under conditions that promote biofilm formation. The major signaling pathways directing hyphal guidance and biofilm construction are the Ras1-cAMP-Efg1 and MAP kinase pathways [25,34,35]. Interestingly, *L. muscari* significantly reduced the expression levels of RAS1, CYR1, and EFG1, which are part of the Ras1-cAMP-Efg1 pathway. The Ras1p GTPase activates Cyr1p adenylate cyclase to produce cAMP, leading to the activation of the Efg1p transcription factor. This factor significantly influences the regulation of several gene expressions related to hyphae, such as ALS3, HWP1, and ECE1, which are crucial for *C. albicans* adherence [36] and biofilm formation [35,37,38,39]. Consequently, the transcription of ALS3, HWP1, and ECE1 was also repressed.

In contrast, *L. muscari* did not alter the expression levels of HST7 and TEC1 genes. HST7 is responsible for encoding a protein kinase within the MAP kinase pathway, whereas TEC1 encodes a transcription factor that independently enhances the expression of genes specific to hyphal formation, separate from the two pathways described above [40].

While the exact components of the extracellular matrix in *C. albicans* biofilms remain not fully characterized, its known components include carbohydrates, proteins, and nucleic acids as previously described [41]. The major extracellular carbohydrate, β-1,3-glucan, produced by the enzyme β-1,3-glucan synthase (Gsc1p), led to the inclusion of the GSC1 transcript as a marker for matrix production [42]. As expected, *L. muscari* downregulated GSC1 gene expression. The protein Zap1p (zinc-responsive activator protein) in *C. albicans*, which responds to zinc levels, promotes cell dispersal by downregulating matrix production. Zap1p directly activates the expression of CSH1 and IFD6, which suppresses substrate accumulation and indirectly inhibits the expression of other alcohol dehydrogenase genes, such as ADH5, which are involved in substrate generation [43]. *L. muscari* reduced the expression of ZAP1 and CSH1 yet had no impact on ADH5 expression. It is worth noting that ZAP1is also essential for the proper morphogenesis of *C. albicans*, aligning with observations that *C. albicans* ZAP1 mutants predominantly exhibit yeast growth in biofilms [43,44]. Consistently, microscopic examination showed that *C. albicans* cells treated with *L. muscari* predominantly exhibited yeast growth after 24 h of incubation.

The identification of chemical components in *L. muscari* that possess anti-biofilm activity is a subject of our future research. Previous studies have indicated that the extract of *L. muscari*, particularly the ethanol extract, exhibits antioxidant, anticancer, and anti-thrombotic properties [22,45,46]. Historically, these extracts have been used as medicinal remedies in East Asia, even before the identification of their effective chemicals. With the advancements in scientific instruments, the bioactive chemical compositions of these extracts have been recently reported. For instance, DT-13 (25 (*R, S*)-ruscogenin-1-*O*-[β-d-glucopyranosyl—(1→2)] [β-d-xylopyranosyl-(1→3)]-β-d-fucopyranoside), the most extensively examined chemical in *L. muscari*, has been reported to exhibit all three aforementioned activities and is known to downregulate matrix metalloproteinases and p38 activation in cancer cells. Two other compounds, norcurlignan ((2*S*,3*R)*-methyl-7-hydroxy-2-(4-hydroxy-3-methoxy-phenyl)-3-(hydroxymethyl)-2,3-dihydrobenzofuran-5-carboxylate) and limlactone ((4R,5S)-5-(3-hydroxy-2,6-dimethylphenyl)-4-isopropyldihydrofuran-2-one) were also identified in the 80% ethanol extract of *L. muscari* and both demonstrated antioxidant activities. However, none of the of *L. muscari* have been reported to inhibit the biofilm formation of *C. albicans*. Given the increasing number of reported cases of antifungal-resistant *C. albicans*, it is worthwhile to investigate the potential anti-biofilm components of *L. muscari* [47].

These findings suggest that *L. muscari* represses the formation of *C. albicans* biofilms by influencing the yeast–hyphal transition. Consequently, *L. muscari* shows promise as a potential coating agent for prosthetic medical devices as other antifungal treatments similarly do. Additionally, preclinical in vivo animal experiments should be performed to verify the possibility that *L. muscari* can be used as an agent to increase antifungal activity. To optimize the use of *L. muscari* in clinical application, studies should also be undertaken to identify a suitable solvent substitute with adequate solvency.

## 4. Materials and Methods

### 4.1. Strains

The *C. albicans* used in this study, KCTC 7965, was obtained from the Korean Collection for Type Cultures (Korean Collection for Type Cultures, Daejeon, Republic of Korea). The strain was preserved in 20% glycerol (BD Difco, NJ, USA) at −70 °C and cultured on YPD plates [peptone 20 g/L (BD Difco, Franklin Lakes, NJ, USA), yeast extract 10 g/L (BD Difco, NJ, USA), and 2% glucose (*w/v*) (Daejung, Siheung, Republic of Korea)].

### 4.2. Sample Preparation

The leaves of *L. muscari* were collected in Jeju, Republic of Korea (GPS: 33.3617° N, 126.5292° E). These leaves were thoroughly rinsed with distilled water and subsequently freeze-dried in preparation for extraction. A total of 100 g of the freeze-dried leaves of *L. muscari* were subjected to extraction in 1 L of 80% ethanol for 24 h. The resulting extracts were concentrated using a rotary evaporator (EYELA, Tokyo, Japan) which was connected to a refrigerated circulating bath (Jeio Tech, Seoul, Republic of Korea). This concentrated extract was then filtered using NO.2 filter paper (WHATMAN, Buckinghamshire, UK). The filtered products were once again freeze-dried and subsequently dissolved in 99.0% DMSO (Junsei, Tokyo, Japan) to achieve a concentration of 10 mg/mL. It is important to note that for all the negative controls utilized in this study, an equivalent volume of 99.0% DMSO was used since the influence of DMSO on *C. albicans* was previously reported [48]. The final DMSO concentration for each sample was 0.25%.

### 4.3. Inhibition of Biofilm Formation

The formation of biofilm was detected using the crystal violet assay [49]. *C. albicans* at a concentration of 5 × 10^5^ CFU/mL was added to the wells of a 96-well plate (SPL, Pocheon, Republic of Korea), mixed in RPMI 1640 medium (Welgene, Gyeongsan, Republic of Korea), and *L. muscari* extract, ranging from 1.56 to 25 μg/mL, was added, and the mixture was cultured at 37 °C for 24 h. The plates were then incubated at 30 °C for 24 h. Each experiment included growth control and sterilized media control wells. After three washes with 200 μL of PBS, 100 μL of 1% aqueous crystal violet solution was added and incubated for 30 min. After staining, each well was washed three times with 200 μL of PBS and immediately destained with 150 μL of 30% acetic acid (Daejung, Siheung, Republic of Korea) for 15 min. The absorbance at 595 nm was measured using a microplate reader (BioTek Instruments, Seoul, Republic of Korea). Biofilm formation was normalized to the control using the following formula: [(1 − (OD_595_DMSO − OD_595_ compound)/OD_595_DMSO) × 100%] (Figure 1).

### 4.4. Combined Antifungal Effect of L. muscari Extract and Antifungal Agent

The combined antifungal effect of the extract and antifungal agent was measured according to previous work [50]. *C. albicans* was cultured overnight in YPD and then diluted to 1 × 10^6^ cells/mL with fresh YPD in 96-well plates (SPL, Pocheon, Republic of Korea) prior to combinational treatment for biofilm formation. A total of 1 mL of Candida suspension was exposed to various concentrations of *L. muscari*, in combination with miconazole at a concentration of 3.125 μg/mL. The indicated concentrations of *L. muscari* and miconazole alone were also applied for comparison.

Biofilms were positioned at the bottom of the well and incubated at 30 °C for 24 h. After washing with PBS to remove planktonic cells, the cells were sonicated to break up clumps. The counts of biofilm fungal CFU were determined by plating serial dilutions on YPD agar.

### 4.5. Morphological Transition Using RPMI 1640 and 10% FBS YPD Liquid Medium

*C. albicans* was cultured overnight in a YPD medium. A concentration of 1 × 10^6^ cells/mL with or without *L. muscari* extract was cultured in RPMI 1640 medium or 10% FBS YPD medium for 4 h at 37 °C. The inhibition of the transition from yeast to hyphal morphology was quantified by counting the number of individual budding cells relative to the number of hyphae in the population, as previously described [51]. At least 100 cells were counted in triplicate for each well, and all analyses were repeated five times. Images of the cells were captured using a microscope.

### 4.6. Quantitative RT-PCR Analysis

*C. albicans* was cultured on YPD agar. A single colony was selected, stirred at 30 °C, and inoculated into YPD. The following day, cultures at 1 × 10^6^ cells/mL were diluted with YPD. Aliquots of the diluted suspension were treated with *L. muscari* extract at concentrations ranging from 1.56 to 25 μg/mL and incubated at 30 °C on an orbital shaker. Total RNA extraction was performed using TRIzol reagent (Thermo Fisher Scientific, Waltham, MA, USA) according to the manufacturer’s instructions. Reverse transcription was carried out with 1 μg of RNA using reverse transcriptase (NanoHelix, Daejeon, Republic of Korea) to obtain cDNA. Quantitative real-time PCR (qRT-PCR) was conducted using 2X SybrGreen qPCR Master Mix (CellSafe, Suwon, Republic of Korea). Primer sequences used for the amplification of genes including GSP1, RAS1, EFG1, TEC1, CDC35, ALS3, HWP1, and ECE1 are listed in Table 1, which were used in the previous paper [52].

### 4.7. C. albicans Growth Test

The growth curve was obtained with some modifications [53]. Fungal cultures were set up at 1 × 10^6^ cells/mL using fresh YPD broth. After adding *L. muscari* extract, it was cultured at 30 °C. The growth was evaluated by measuring OD_600_ with a microplate reader at intervals of 0, 1, 2, 4, 8, 12, and 24 h.

### 4.8. Statistical Analysis

All experiments were performed at least three times, and data were presented as the ± mean standard deviation (S.D). Statistical analysis was performed using Microsoft Excel 2021 (Microsoft, Redmond, WA, USA) and Statistical Package for the Social Sciences (SPSS) 28.0 (IBM Corp, New York, NY, USA). All graphs except microscopic images were analyzed by Microsoft Excel 2021. Statistical significance was defined at a *p*-value less than 0.05.

## 5. Conclusions

*L. muscari* should be a good candidate for anti-virulence agents against Candida infections because it inhibits biofilm formation and hyphal transition and increases antifungal susceptibility. This antifungal efficacy of *L. muscari* could suggest the direction for the development of new antifungal strategies with a lower virulence of *C. albicans*.

## Figures and Tables

**Table 1:** Primer list used in this paper.

Primers	Primer Sequence	Gene Function
ACT 1	F: TAGGTTTGGAAGCTGCTGG R: CCTGGGAACATGGTAGTAC	Control
ALS 3	F: GGTTATCGTCCATTTGTTG R: TTCTGTATCCAGTCCATCT	Hyphal-specific genes
CYR 1	F: GTTTCCCCCACCACTCA R: TTGCGGTAATGACACAACAG	Ras-cAMP-Efg1 pathway
ECE 1	F: ACAGTTTCCAGGACGCCAT R: ATTGTTGCTCGTGTTGCCA	Hyphal-specific genes
EFG 1	F: TTGAGATGTTGCGGCAGGAT R: ACTGGACAGACAGCAGGAC	Ras-cAMP-Efg1 pathway
GSC 1	F: CCCATTCTCTAGGCACGA R: ATCAACAACCACTTGCTTCG	Extracellular matrix
HST 7	F: GCCAGTATGGTCGGAGGAT R: ACATAGGCATCGTCTTCGTC	MAP kinases pathway
HWP 1	F: ACAGGTAGACGGTCAAGG R: GGGTAATCATCACATGGTTC	Ras-cAMP-Efg1 pathway
RAS 1	F: GAGGTGGTGGTGTTGGTA R: TCTTCTTGTCCAGCAGTATC	Cph2-Tec1 pathway
TEC 1	F: GCACTGGCTTCAAGCTCAAA R: GCTGCTGCACTCAAGTTCTG	Extracellular matrix
ZAP 1	F: ATCTGTCCAGTGTTGTTTGTA R: AGGTCTCTTTGAAAGTTGTG	Extracellular matrix
ADH 5	F: ACCTGCAAGGGCTCATTCTG R: CGGCTCTCAACTTCTCCATA	Extracellular matrix
CSH 1	F: CGTGAGGACGAGAGAGAAT R: CGAATGGACGACACAAAACA	Extracellular matrix

## Data Availability

The data presented in this study are available in article.
